# Genome Assemblies of Two Rare Opportunistic Yeast Pathogens: *Diutina rugosa* (syn. *Candida rugosa*) and *Trichomonascus ciferrii* (syn. *Candida ciferrii*)

**DOI:** 10.1534/g3.119.400762

**Published:** 2019-10-01

**Authors:** Verónica Mixão, Ester Saus, Antonio Perez Hansen, Cornelia Lass-Florl, Toni Gabaldón

**Affiliations:** * Centre for Genomic Regulation, The Barcelona Institute of Science and Technology, Dr. Aiguader 88, Barcelona 08003, Spain,; † Life Sciences Department. Barcelona Supercomputing Center (BSC). Jordi Girona, 29. 08034 Barcelona, Spain,; ‡ Mechanisms of Disease Department. Institute for Research in Biomedicine (IRB). Barcelona, Spain,; § Division of Hygiene and Medical Microbiology, Innsbruck Medical University, Austria,; ** Universitat Pompeu Fabra (UPF), Barcelona, Spain, and; †† ICREA, Pg. Lluis Companys 23, Barcelona 08010, Spain

**Keywords:** *Diutina rugosa*, *Trichomonascus ciferrii*, genome assembly, yeast, pathogen, *Candida rugosa*, *Candida ciferrii*

## Abstract

Infections caused by opportunistic yeast pathogens have increased over the last years. These infections can be originated by a large number of diverse yeast species of varying incidence, and with distinct clinically relevant phenotypic traits, such as different susceptibility profiles to antifungal drugs, which challenge diagnosis and treatment. *Diutina rugosa* (syn. *Candida rugosa*) and *Trichomonascus ciferrii* (syn. *Candida ciferrii*) are two opportunistic rare yeast pathogens, which low incidence (< 1%) limits available clinical experience. Furthermore, these yeasts have elevated Minimum Inhibitory Concentration (MIC) levels to at least one class of antifungal agents. This makes it more difficult to manage their infections, and thus they are associated with high rates of mortality and clinical failure. With the aim of improving our knowledge on these opportunistic pathogens, we assembled and annotated their genomes. A phylogenomics approach revealed that genes specifically duplicated in each of the two species are often involved in transmembrane transport activities. These genomes and the reconstructed complete catalog of gene phylogenies and homology relationships constitute useful resources for future studies on these pathogens.

*Candida* species are the most common cause of hospital-acquired fungal infections, very often leading to patient’s death (Pfaller and Diekema 2007; Lass-Flörl 2009; Brown *et al.* 2012; Gabaldón and Carreté 2016). Although *Candida albicans*, *Candida glabrata* and *Candida parapsilosis* are the species with highest prevalence (Pfaller and Diekema 2007; Jordà-Marcos *et al.* 2007), in the last years the incidence of “rare yeast” infections has increased (Lass-Flörl 2009; Pfaller *et al.* 2012; Bretagne *et al.* 2017). By “rare yeasts” we mean ascomycetous yeasts that have very low prevalence (< 1% of clinical *Candida* infections) and have high Minimum Inhibitory Concentrations (MICs) toward at least one class of antifungal drugs (Guitard *et al.* 2013; Jung *et al.* 2015; Bretagne *et al.* 2017).

*Diutina rugosa* (syn. *Candida rugosa* (Khunnamwong *et al.* 2015)) and *Trichomonascus ciferrii* (syn. *Candida ciferrii* (Kurtzman and Robnett 2007)) are two “rare yeasts” (Pfaller *et al.* 2010). *D. rugosa* has been reported as a causative agent of veterinary infections (Moretti *et al.* 2000; Crawshaw *et al.* 2005; Scaccabarozzi *et al.* 2011), and therefore might have impact in industry and economics. Furthermore, it has been identified as the etiological agent of several clinical infections, including a clinical outbreak in Brazil (Lopes Colombo *et al.* 2003; Pfaller *et al.* 2010). Thus, this species is considered an emerging fungal pathogen (Pfaller *et al.* 2006; Minces *et al.* 2009). Indeed, in a 10-year multi-center study a 10-fold increase in the number of *D. rugosa* clinical cases was reported (Pfaller *et al.* 2010). *T. ciferrii* has also been reported as an opportunistic pathogen in some sporadic cases of infections in immunocompromised patients (Gunsilius *et al.* 2001; Pfaller *et al.* 2010; Saha *et al.* 2013; Villanueva-Lozano *et al.* 2016; Upadhyay *et al.* 2018). Both species were recently shown to present high MICs to azoles and echinocandins (Pfaller *et al.* 2006; Pérez-Hansen *et al.* 2019).

Next Generation Sequencing (NGS) is a powerful tool to study the genomic background of pathogens, which might reveal many of their features. In the last years, more and more studies performing NGS analysis on yeast pathogens were published and showed the relevance of whole-genome sequence for the study of pathogenic genomic determinants (Butler *et al.* 2009; Pryszcz *et al.* 2015; Schröder *et al.* 2016; Ropars *et al.* 2018; Mixão *et al.* 2019). In this context, we decided to sequence the genome of both *D. rugosa* and *T. ciferrii*, which will be useful for future studies on these opportunistic pathogens.

## Materials and Methods

### Library preparation and genome sequencing

We sequenced the type strains for *D. rugosa* (CBS613) and *T. ciferrii* (CBS4856). Genomic DNA extraction was performed using the MasterPure Yeast DNA Purification Kit (Epicentre, United States) following manufacturer’s instructions and all reagents mentioned are from the kit if not specified otherwise. Briefly, cultures were grown in an orbital shaker overnight (200 rpm, 30°) in 15 ml of YPD medium (Yeast extract-Peptone-Dextrose medium: 10 g of yeast extract, 20 g of bacto peptone and 50 ml of dextrose 40% in 1 L of distilled water). Cells were harvested using 4.5 ml of each culture by centrifugation at maximum speed for 2 min, and then they were lysed at 65° for 15 min with 300 μl of yeast cell lysis solution (containing 1 μl of RNAse A). After being on ice for 5 min, 150 μl of MPC protein precipitation reagent were added into the samples, and they were centrifuged at 16.000 g for 10 min to pellet the cellular debris. The supernatant was transferred to a new tube, DNA was precipitated using 100% cold ethanol and centrifuging the samples at 16.000 g, 30 min, 4°. The pellet was washed twice with 70% cold ethanol and, once the pellet was dried, the sample was resuspended in 100 μl of TE. All gDNA samples were cleaned to remove the remaining RNA using the Genomic DNA Clean & Concentrator kit (Epicentre) according to manufacturer’s instructions. Total DNA integrity and quantity of the samples were assessed by means of agarose gel, NanoDrop 1000 Spectrophotometer (Thermo Fisher Scientific, United States) and Qubit dsDNA BR assay kit (Thermo Fisher Scientific).

Whole-genome sequencing was performed at the Genomics Unit from Centre for Genomic Regulation (CRG) with an Illumina HiSeq2500 machine. Libraries were prepared using the NEBNext Ultra DNA Library Prep kit for Illumina (New England BioLabs, United States) according to manufacturer’s instructions. All reagents subsequently mentioned are from the NEBNext Ultra DNA Library Prep kit for Illumina if not specified otherwise. 1 μg of gDNA was fragmented by nebulization using the Covaris S2 instrument (Covaris Inc.) to a size of ∼600 bp. After shearing, the ends of the DNA fragments were blunted with the End Prep Enzyme Mix, and then NEBNext Adaptors for Illumina were ligated using the Blunt/TA Ligase Master Mix. The adaptor-ligated DNA was cleaned-up using the MinElute PCR Purification kit (Qiagen, Germany) and a further size selection step was performed using an agarose gel. Size-selected DNA was then purified using the QIAgen Gel Extraction Kit with MinElute columns (Qiagen) and library amplification was performed by PCR with the NEBNext Q5 Hot Start 2X PCR Master Mix and index primers (12–15 cycles). A further purification step was done using AMPure XP Beads (Agentcourt, United States). Final libraries were analyzed using Agilent DNA 1000 chip (Agilent) to estimate the quantity and check size distribution, and they were then quantified by qPCR using the KAPA Library Quantification Kit (KapaBiosystems, United States) prior to amplification with Illumina’s cBot. Libraries were loaded and sequenced in paired-end reads of 125bp on Illumina’s HiSeq2500. Base calling was performed using Illumina pipeline software. In multiplexed libraries, we used 6 bp internal indexes (5' indexed sequences). De-convolution was performed using the CASAVA software (Illumina, United States). Sequence data has been deposited in short read archive (SRA) under the BioProject Accession No. PRJNA531406.

### De novo genome assembly and phylome reconstruction

Raw sequencing data were inspected with FastQC v0.11.5 (http://www.bioinformatics.babraham.ac.uk/projects/fastqc/). Paired-end reads were filtered for quality below 10 or size below 31 bp and for the presence of adapters with Trimmomatic v0.36 (Bolger *et al.* 2014). The K-mer Analysis Toolkit v2.4.1 (KAT; (Mapleson *et al.* 2017)) was used to get the GC content and k-mer frequency distribution and estimate the expected genome size. SOAPdenovo v2.04 (Luo *et al.* 2012) was used to perform genome assembly. Redundant contigs were removed with Redundans v0.13c (Pryszcz and Gabaldón 2016) using default parameters, *i.e.*, 51% minimum identity and at least 80% overlap. The quality of the assembly was inspected with Quast v4.5 (Gurevich *et al.* 2013) and KAT v2.4.1 (Mapleson *et al.* 2017). Species identification was confirmed by BLASTn (Zhang *et al.* 2000) of the respective ITS region (accession: NR_111249.1 for *D. rugosa* and NR_111160.1 for *T. ciferrii*), as recommended (Stavrou *et al.* 2018). Genome annotation was performed with Augustus v3.1 (Stanke and Morgenstern 2005), using *Meyerozyma guilliermondii* and *Sacharomyces cerevisiae* as model organisms for *D. rugosa* and *T. ciferrii*, respectively. The Ascomycota dataset in BUSCO v3 (Waterhouse *et al.* 2018) was used to assess completeness.

Phylome reconstruction - *i.e.*, the complete collection of phylogenies for every gene encoded in the genome - was performed using the PhylomeDB pipeline (Huerta-Cepas *et al.* 2014), as described in (Pryszcz *et al.* 2015), considering twenty-seven species (Supplementary file 1). This was done for both *D. rugosa* and *T. ciferrii*, using their respective predicted proteomes as seed. These phylomes and the corresponding orthology and paralogy relationships are available for browsing or download in PhylomeDB (Huerta-Cepas *et al.* 2014) with ID 932 and 842, respectively. Gene gain and loss analysis in seed branch was performed based on the phylome results. A BLASTp (Zhang *et al.* 2000) was performed against the UniProt database (UniProt Consortium 2019) (accessed on April 30^th^, 2019), in order to determine the possible function associated with these genes, as well as their GO terms. An enrichment analysis was done using FatiGO (Al-Shahrour *et al.* 2007). Species-tree reconstruction was based on the final concatenated alignment of 469 single genes, comprising 297,788 amino-acid positions, with RAxML v8.2.4 (Stamatakis 2014), using the PROTGAMMALG substitution model and performing rapid bootstrapping with 1000 replicates. A BLASTp (Zhang *et al.* 2000) of the species-specific genes against the non-redundant database maintained by NCBI (NCBI Resource Coordinators 2015) (accessed on September 13^th^, 2019), considering only hits with e-value < 0.001 and query coverage > 50%, was performed to determine whether these genes have homologs in species which were not considered for phylome reconstruction.

### Read mapping and variant calling

Read mapping for all strains (Table 1) was performed with BWA-MEM v0.7.15 (Li 2013). Picard v2.1.1 (http://broadinstitute.github.io/picard/) was used to sort the resulting file by coordinate, as well as to mark duplicates, create the index file, and obtain mapping statistics. Mapping results were inspected with IGV version 2.0.30 (Thorvaldsdóttir *et al.* 2013). Mapping coverage was determined with SAMtools v0.1.18 (Li *et al.* 2009).

**Table 1:** Metrics of *D. rugosa* and *T. ciferrii* nuclear genome assemblies, with indication of their respective genome size, N50, GC content, coverage, percentage of mapped reads, variants per kilo-base (kb) and heterozygous (heter) variants per kb

Species (strain)	Size (Mb)	N50	GC (%)	Coverage (reads/position)	Mapped reads (%)	SNPs/kb	Heter SNPs/kb
*Diutina rugosa* (CBS613)	13.4	193 138	49.56%	175.9	64.07%	0.09	0.07
*Trichomonascus ciferrii* (CBS4856)	20.5	69 012	47.46%	209.6	98.35%	0.12	0.09

Samtools v0.1.18 (Li *et al.* 2009) and Picard v2.1.1 (http://broadinstitute.github.io/picard/) were used, respectively, to index the reference and create a dictionary to be used in subsequent variant calling steps. GATK v3.6 (McKenna *et al.* 2010) was used to call and filter variants with the tools HaplotypeCaller and VariantFiltration, respectively, as described by (Mixão *et al.* 2019). In order to determine the number of SNPs/kb, a file containing only SNPs was generated with the SelectVariants tool. Moreover, for this calculation only positions in the reference with 20 or more reads were considered for the genome size, and these were determined with bedtools genomecov v2.25.0 (Quinlan and Hall 2010).

### Mitochondrial genome assembly

NOVOPlasty v2.7.2 (Dierckxsens *et al.* 2017) with default parameters was used to assemble *D. rugosa* and *T. ciferrii* mitochondrial genomes, taking as seed input the respective *Cox2* gene (accession numbers: KT832772.1 and DQ443088.1, respectively). The final assemblies were complete, as the assembly program was able to circularize each of them. Mitochondrial genome annotation was performed with MITOS2 (Bernt *et al.* 2013). Read mapping to these mitochondrial assemblies was performed as mentioned before for the nuclear genome.

### Data availability

Data generated by this project can be found under the BioProject PRJNA531406, including sequencing data, genome assemblies and respective annotation. Phylomes can be found in PhylomeDB, with the phylome IDs 842 and 932. A list of species used for phylome reconstruction, and the results of the enrichment analysis can be found in Supplementary files 1 and 2, respectively. Plots related to the *k*-mer analysis in the genome assemblies are in Supplementary figure 1. Supplemental material available at figshare: https://doi.org/10.25387/g3.8945048.

## Results and Discussion

### Genome sequencing and assembly

In this study we sequenced the type strains of *D. rugosa* and *T. ciferrii*, using an Illumina-based, pair-end sequencing strategy (see Materials and Methods). GC content and 27-mer count analyses of the sequencing reads revealed only one peak for each strain (Supplementary figure 1), suggesting that the two sequenced strains are highly homozygous. Based on the same 27-mer counts, we estimated genome sizes of approximately 13 Mb and 19 Mb for *D. rugosa* and *T. ciferrii*, respectively (see Materials and Methods). We next performed a *de novo* genome assembly for each of these species (see Materials and Methods). The final nuclear genome assembly of *D. rugosa* comprised 13.4 Mb, with 49.56% GC content and a N50 of 193,138 bp (Table 1). This assembly was divided in 171 contigs, of which 88 were longer than 25 kb, representing 97.7% of the genome. Automated gene prediction resulted in 5,821 protein-coding genes (see Materials and Methods). Despite the fact that the genome size was close to our estimations and similar to the one reported for the closely-related species *Diutina catenulata* (13.1 Mb), the number of predicted proteins was substantially lower than the 7,128 proteins annotated in the close relative *D. catenulata* (O’Brien *et al.* 2018). Furthermore, only 64.07% of the reads could be mapped to *D. rugosa* nuclear genome assembly. These observations made us question the completeness of our assembly. However, KAT (Mapleson *et al.* 2017) reported that 98.96% of 27-mers was represented in the assembly (Supplementary figure 1), and BUSCO (Waterhouse *et al.* 2018) reported 97.7% completeness of *D. rugosa* predicted proteome. Finally, most of the reads that did not map to the nuclear genome were found to correspond to the mitochondrial genome (see section “Mitochondrial genome assembly”). Thus, we consider that *D. rugosa* genome annotation is not significantly underestimating its gene content. It remains to be investigated whether the large number of proteins reported for *D. catenulata* is an annotation artifact or a real biological difference. There are no other available genomes for this genus, and the close relatives *M. guilliermondii* and *Scheffersomyces stipitis* have 5,920 and 5,841 annotated proteins, respectively (Jeffries *et al.* 2007; Butler *et al.* 2009).

The nuclear genome assembly of *T. ciferrii* comprised 20.5 Mb, with 47.46% GC content and a N50 of 60,012 bp (Table 1). This assembly entailed 584 contigs, of which 132 were longer than 25 kb, representing 84.3% of the genome. Genome annotation predicted 6,913 proteins (see Materials and Methods). To the best of our knowledge, there is no other genome assembly of the *Trichomonascus* genus published so far, which would allow us to have a better assessment of the quality of our assembly. Even so, 27-mer frequency analysis showed that 99.83% of *T. ciferrii* 27-mers was represented in the assembly (Supplementary figure 1), and BUSCO (Waterhouse *et al.* 2018) estimated 93.4% proteome completeness, suggesting a good representation of the *T. ciferrii* genome in our assembly. Read mapping and variant calling confirmed that both *D. rugosa* and *T. ciferrii* are highly homozygous, having 0.07 and 0.09 heterozygous SNPs/kb, respectively (Table 1). It is worth mentioning that both genomes present homozygous SNPs (0.02 SNPs/kb in *D. rugosa* and 0.03 SNPs/kb in *T. ciferrii*), which is unexpected as the reads were mapped on the respective assembly. This situation can probably be a result of errors introduced during the sequencing process or data analysis (*i.e.*, read assembly, read mapping or variant calling).

### Mitochondrial genome assembly

**Figure 1:**
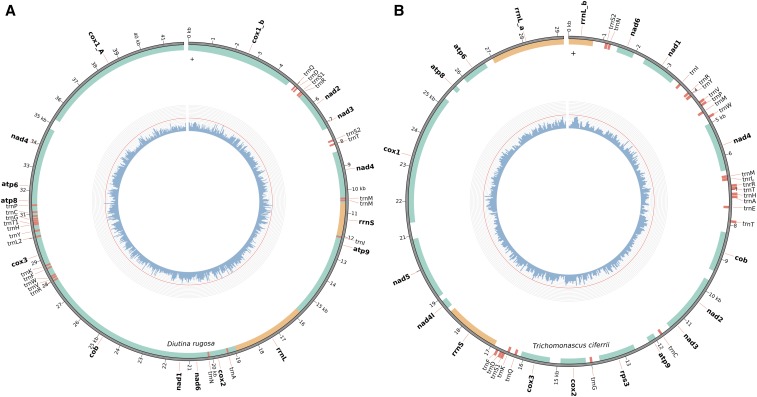
Mitochondrial genome representation of A) *D. rugosa* and B) *T. ciferrii*. Protein-coding genes are marked in green, tRNA genes are marked in red, and ribosomal genes are marked in orange. The blue histogram in the center represents the GC content variation.

As mentioned before, only 64.07% of *D. rugosa* sequencing reads mapped to the respective genome assembly. Thus, we decided to assemble its mitochondrial genome (see Materials and Methods), in order to see whether the remaining reads could come from it. A final 41.8 kb circular mitochondrial genome assembly was obtained, suggesting that the assembly is complete (Figure 1a). Read mapping confirmed that 34.5% of *D. rugosa* sequencing reads corresponded to the mitochondrial genome, which suggests a high mitochondrial content in this yeast. We have assembled *T. ciferrii* mitochondrial genome as well, obtaining a circular assembly with 29.2 kb (Figure 1b), where 2.2% of *T. ciferrii* sequencing reads mapped. While we annotated 14 protein-coding genes in *D. rugosa* mitochondria, in *T. ciferrii* we annotated 16. The major difference between the two species involved the *nad4L* (associated to complex I) and *rps3* genes, which were absent in *D. rugosa*.

### Comparative genomics

**Figure 2:**
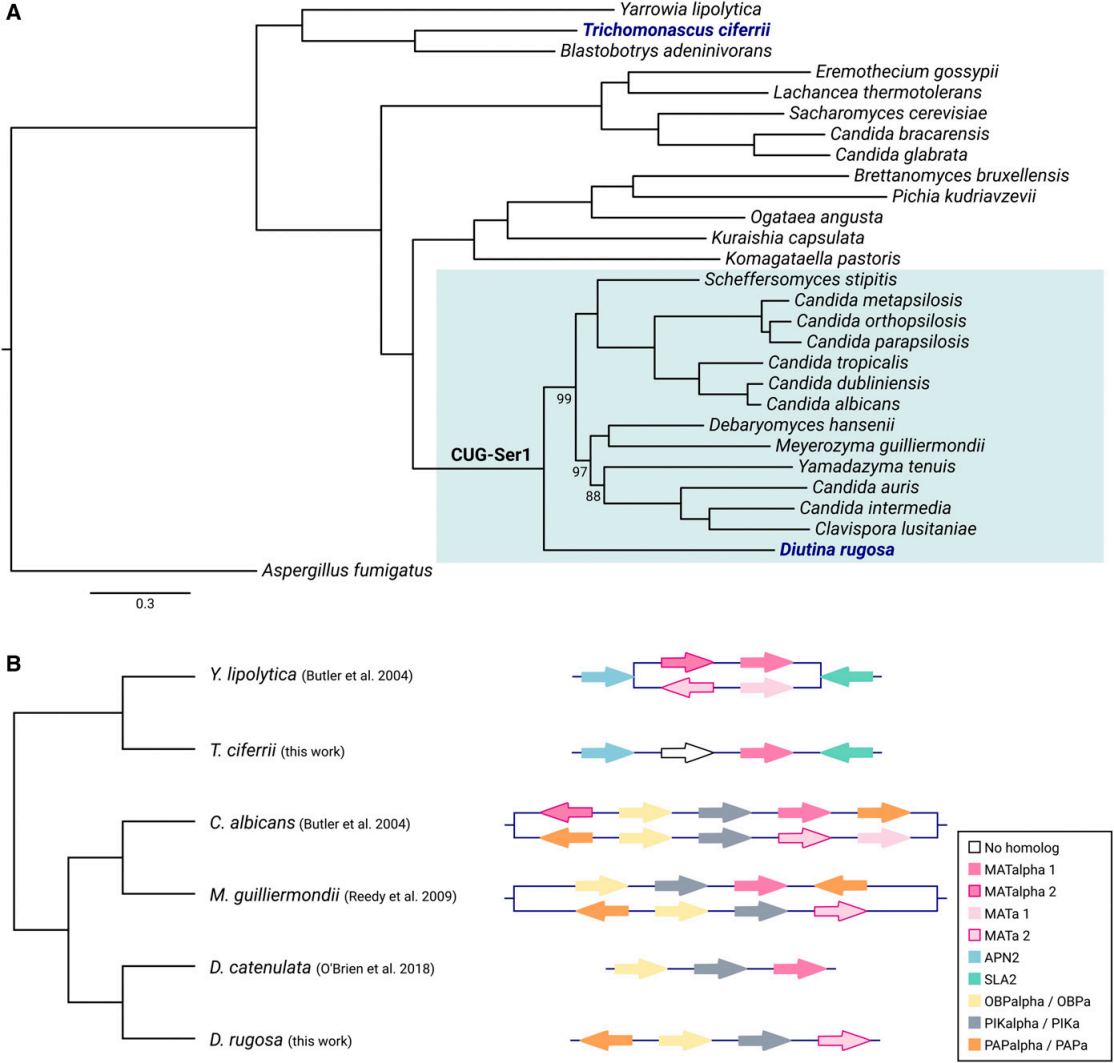
Comparative genomics of *D. rugosa* and *T. ciferrii* genomes. A) Maximum Likelihood phylogenetic tree of the concatenated alignment of 469 single genes, comprising 297,788 amino-acid positions. When the branch support is different from 100 the values are presented close to the respective branch. CUG-Ser1 clade is highlighted in blue. *D. rugosa* and *T. ciferrii* are marked in dark blue and bold. B) Schematic representation of the *MAT* locus of *D. rugosa* and *T. ciferrii* in comparison with closely-related species. The tree presents their phylogenetic relationship, but the branch length does not correspond to their phylogenetic distance. Each arrow represents a different gene with the color indicating the gene name.

In order to elucidate particular characteristics of *D. rugosa* and *T. ciferrii* we decided to follow a comparative genomics approach, and compared their nuclear genomes/proteomes with other species. We reconstructed the complete collection of gene evolutionary histories (*i.e.*, the phylome) (Gabaldón 2008) for each of these two species, in the context of twenty-six other species (see Material and Methods, Supplementary file 1). We identified 770 species-specific genes for *D. rugosa* and 1,217 for *T. ciferrii*, from which only 247 and 391, respectively, had homologs in species which were not considered for phylome reconstruction (see Materials and Methods). In both species, species-specific genes were not enriched in any particular function. Interestingly, genes specifically duplicated in each of the two species seemed to be enriched in transmembrane transport activities, as well as, oxidoreductase activity (detailed information can be found in Supplementary file 2). As can be observed in the species tree (Figure 2a), *D. rugosa* belongs to the CUG-Ser1 clade, while *T. ciferrii* is close to *Yarrowia lipolytica*. This shows that although very distantly related these two emergent pathogens present gene duplications affecting similar functions. Furthermore, in the case of *D. rugosa*, it is worth noting an enrichment in aspartic-type endopeptidase activity and ferrichrome transporter activity (Supplementary file 2), as both have been reported as important for pathogenic behavior, particularly in *Candida* species (Heymann *et al.* 2002; Naglik *et al.* 2003).

An earlier study on *D. catenulata* genome revealed an interesting break in the *MAT* locus of this species (O’Brien *et al.* 2018). By comparison with the *MAT* locus of *M. guilliermondii* (Reedy *et al.* 2009), these authors reported the absence of *PAP* gene close to *MAT alpha1* (Figure 2b), being the *PAP* gene instead in a different contig of *D. catenulata* genome assembly (O’Brien *et al.* 2018). Furthermore, they found that this gene was phylogenetically closer to *PAP a* than to *PAP alpha* (O’Brien *et al.* 2018). To assess whether this characteristic is shared within the *Diutina* genus, we here inspected the *MAT* locus of *D. rugosa*. Contrary to *D. catenulata* (O’Brien *et al.* 2018), *D. rugosa MAT* locus corresponded to the *MAT a* allele, where, similarly to *M. guilliermondii*, we could only find *MAT a2* (Figure 2b). Moreover, when comparing to *M. guilliermondii* (Reedy *et al.* 2009), we could not identify any particular rearrangement in this locus. Regarding the *MAT* locus of *T. ciferrii*, we observed that, in contrast to *Y. lipolytica* where both *MAT a* and *MAT alpha* were described (Butler *et al.* 2004), it only presents the *MAT alpha* allele (Figure 2b). It is worth to mention that although there is a protein-coding gene in the place of *MAT alpha2*, this protein does not present any homolog and therefore we were only able to identify *MAT alpha1* in *T. ciferrii* genome (Figure 2b).

### Concluding remarks

We have here reported the genomes of two emergent yeast pathogens, which are phylogenetically very distantly related, namely *D. rugosa* and *T. ciferrii*. These two reference genomes provide an important resource for the assessment of relevant aspects of these yeasts, including the genetic bases of their clinically relevant traits, as virulence and drug resistance. In addition, the two phylomes, which include a full repertoire of gene evolutionary histories and a catalog of orthologs and paralogs, can be used to trace the origin and evolution of genes of interest. Therefore, the data provided by this publication will certainly be of interest for the study of emergent yeast pathogens.
